# Giant Effective Damping of Octupole Oscillation in an Antiferromagnetic Weyl Semimetal

**DOI:** 10.1002/smsc.202000062

**Published:** 2021-04-15

**Authors:** Shinji Miwa, Satoshi Iihama, Takuya Nomoto, Takahiro Tomita, Tomoya Higo, Muhammad Ikhlas, Shoya Sakamoto, YoshiChika Otani, Shigemi Mizukami, Ryotaro Arita, Satoru Nakatsuji

**Affiliations:** ^1^ The Institute for Solid State Physics The University of Tokyo Kashiwa Chiba 277-8581 Japan; ^2^ Trans-scale Quantum Science Institute The University of Tokyo Bunkyo Tokyo 113-0033 Japan; ^3^ CREST Japan Science and Technology Agency (JST) Kawaguchi Saitama 332-0012 Japan; ^4^ Frontier Research Institute for Interdisciplinary Sciences (FRIS) Tohoku University Sendai Miyagi 980-8578 Japan; ^5^ Advanced Institute for Materials Research (AIMR) Tohoku University Sendai Miyagi 980-8577 Japan; ^6^ Center for Spintronics Research Network (CSRN) Tohoku University Sendai Miyagi 980-8577 Japan; ^7^ Department of Applied Physics The University of Tokyo Tokyo 113-8656 Japan; ^8^ Department of Physics The University of Tokyo Tokyo 113-0033 Japan; ^9^ RIKEN, Center for Emergent Matter Science (CEMS) Wako Saitama 351-0198 Japan; ^10^ Center for Science and Innovation in Spintronics (CSIS) Tohoku University Sendai Miyagi 980-8577 Japan

**Keywords:** antiferromagnetic spintronics, damping, Mn_3_Sn, spin dynamics, Weyl semimetal

## Abstract

A magnetic Weyl semimetal is a recent focus of extensive research as it may exhibit large and robust transport phenomena associated with topologically protected Weyl points in momentum space. Since a magnetic texture provides a handle for the configuration of the Weyl points and its transport response, understanding of magnetic dynamics forms the basis for future control of a topological magnet. Mn_3_Sn is an example of an antiferromagnetic Weyl semimetal that exhibits a large response comparable to the one observed in ferromagnets despite a vanishingly small magnetization. The noncollinear spin order in Mn_3_Sn can be viewed as a ferroic order of cluster magnetic octupole and breaks the time‐reversal symmetry, stabilizing Weyl points and the significantly enhanced Berry curvature near the Fermi energy. Herein, the first observation of time‐resolved octupole oscillation in Mn_3_Sn is reported. In particular, the giant effective damping of the octupole dynamics is found, and it is feasible to conduct an ultrafast switching at <10 ps, a hundred times faster than the case of spin‐magnetization in a ferromagnet. Moreover, high domain wall velocity over 10 km s^−1^ is theoretically predicted. This work paves the path toward realizing ultrafast electronic devices using the topological antiferromagnet.

## Graphical abstract

The first observation of time‐resolved oscillation of the cluster magnetic octupole in the magnetic Weyl semimetal Mn_3_Sn is reported. It is found that the dynamics is enhanced by exchange interaction, and it is feasible to conduct an ultrafast switching at <10 ps. This work paves the path toward realizing ultrafast electronic devices using a topological antiferromagnet.

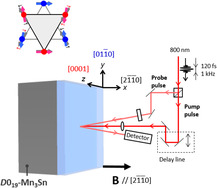

## 1 Introduction

A Weyl semimetal is a topological system in three dimensions, which appears in either time‐reversal‐symmetry (TRS) or inversion‐symmetry broken state.^[^ ^1^, ^2^, ^3^, ^4^ ^]^ It is characterized by gapless electronic excitations called Weyl fermions, formed as a result of a linear crossing of two nondegenerate bands at a pair of momentum points with different chiralities. As these Weyl points can be viewed as unit‐size monopole of underlying Berry curvature, Weyl semimetals may exhibit various exotic phenomena such as Fermi arc, anomalous Hall and Nernst effects (AHE and ANE), chiral anomaly, and gyrotropic effects.^[^ ^1^, ^2^, ^3^, ^4^, ^5^, ^6^, ^7^, ^8^, ^9^, ^10^, ^11^, ^12^, ^13^, ^14^, ^15^, ^16^, ^17^, ^18^ ^]^ In particular, for TRS broken or magnetic Weyl semimetals, magnetic order determines the configuration of the Weyl points in momentum space and provides the handle for controlling the transport responses.^[^ ^10^, ^12^, ^13^, ^14^, ^15^, ^16^, ^17^, ^18^ ^]^ Therefore, understanding of the magnetic dynamics forms the basis for future research and application of Weyl semimetals.

In the field of spintronics, antiferromagnetic (AF) metals have attracted significant attention as next‐generation active materials of electronic devices for their vanishingly small stray field perturbing neighboring cells. The recent rapid development in AF spintronics^[^ ^19^, ^20^, ^21^ ^]^ has led to the demonstration of electric reading and writing of an AF state,^[^ ^22^, ^23^ ^]^ which has been recently further supported by several kinds of AF domain imaging techniques.^[^ ^24^, ^25^, ^26^, ^27^, ^28^ ^]^ Because of its vanishingly small magnetization, the detection means for such an AF metallic state have been restricted to anisotropic magnetoconductance,^[^ ^22^, ^23^ ^]^ quadratic magneto‐optical effects,^[^ ^29^ ^]^ and resonant X‐ray diffraction,^[^ ^30^ ^]^ which are far weaker than the magnetization ***M***‐linear response such as AHE and magneto‐optical effects used for ferromagnets.

**Figure 1:**
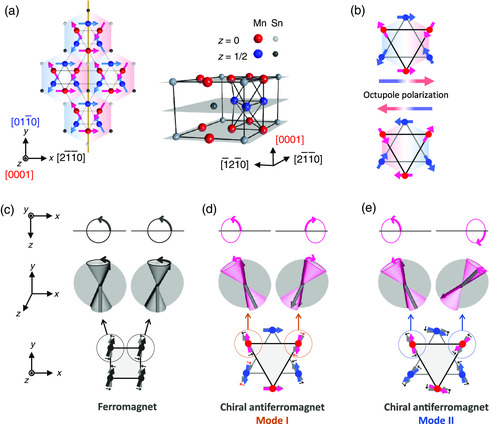
Cluster magnetic octupole and its oscillation modes in *D*0_19_‐Mn_3_Sn. a) Spin and crystal structures of Mn_3_Sn. Different colors are used to denote Mn and Sn atoms in the *z* = 0 and *z* = 1/2 planes. The inverse triangular spin‐structure can be viewed as a ferroic ordering of cluster magnetic octupole, which possesses the same symmetry as the spin‐magnetization (e.g., as indicated by a mirror plane [orange line]). b) Magnetic cluster octupole units with different octupole polarization directions. c) Spin dynamics in a ferromagnet with uniaxial magnetic anisotropy (*K*). Resonant frequency (*ħω*) and typical switching time (Δ*ω*)^−1^ can be ≈*K* and ≈(2*αK*)^−1^ *ħ*, respectively, where *α* is the Gilbert damping constant. d,e) Spin dynamics in a chiral antiferromagnet. Spin‐wave modes I and II correspond to optical and collective precession‐like motions and possess resonant frequencies of *ω*_I_ ≈ $\sqrt{JD}\hbar^{-1}$ and *ω*_II_ ≈ $\sqrt{KJ}\hbar^{-1}$, respectively. *D* and *J* are Dzyaloshinskii–Moriya interaction and exchange interaction energy scales, respectively. Typical switching time for both modes can be estimated as (Δ*ω*_I_)^−1^ ≈ (Δ*ω*_II_)^−1^ ≈ (*αJ*)^−1^ *ħ*.

With this respect, recently discovered *D*0_19_‐Mn_3_Sn stands out as a unique antiferromagnet that exhibits large electric and optical ***M***‐linear responses such as AHE,^[^ ^10^ ^]^ ANE,^[^ ^13^, ^14^ ^]^ and magneto‐optical Kerr effect (MOKE)^[^ ^31^ ^]^ even though it has only a vanishingly small magnetization. Significantly, it has been clarified that Mn_3_Sn is a magnetic Weyl semimetal.^[^ ^10^, ^13^, ^15^ ^]^ The noncollinear chiral magnetic texture in Mn_3_Sn can be viewed as a ferroic order of a cluster magnetic octupole and breaks TRS macroscopically.^[^ ^32^ ^]^ **Figure** **1**a shows the crystal and magnetic structures of Mn_3_Sn. The magnetic moments of Mn lie in the (0001) plane and form an inverse triangular spin structure. In this structure, one unit of the cluster magnetic octupole is made of the six neighboring moments on an octahedron (bilayered triangle) of Mn atoms as featured by a colored hexagon in Figure 1a. By 180° rotation of each spin, the octupole polarization may reverse its direction (Figure 1b). As a result, the in‐plane noncollinear spin order can be viewed as *Q* = 0 order of the magnetic octupole. The AF spin texture with this ferroic order induces large Berry curvature due to Weyl points in momentum space,^[^ ^13^, ^15^ ^]^ leading to the large transverse response. In this regard, the observation of the spin dynamics in a topological magnet would be a key step for future manipulation of the Weyl points in the momentum space and the associated large responses. In addition, similar to the case of the spin‐magnetization dynamics in ferromagnetic metals, understanding and control of the time dependence of the cluster magnetic octupole in a chiral AF metal would be an important step for developing the device physics in magnetism. To date, there has been no report on the time‐resolved observation of spin dynamics in either ferromagnetic or AF Weyl semimetals. Even when we focus on the previous research on antiferromagnets,^[^ ^29^, ^30^, ^33^, ^34^, ^35^, ^36^, ^37^ ^]^ the direct, time‐resolved observation of the spin precession has been limited to AF insulators^[^ ^35^, ^36^, ^37^ ^]^ and never been made in AF metals. A few articles report the time‐resolved dynamics of the order parameter in AF metals^[^ ^29^, ^30^, ^34^ ^]^ but never been able to associate them with the spin‐wave modes. In this article, we report our observation of the time‐resolved spin dynamics in the AF Weyl semimetal Mn_3_Sn.

## 2 Spin Dynamics of an Antiferromagnet

Let us first discuss the case of collective spins in ferromagnetic materials, where each spin is coupled in parallel by exchange interaction (Supporting Information 1). A precessional motion of each spin is always in‐phase as schematically shown in Figure 1c, and thus, the energy scale of the resonant frequency (*ħω* ≈ *K*) is independent of the exchange interaction and is determined by *K* only, where *K* is magnetic anisotropy energy mainly originating from on‐site spin‐orbit interaction. A typical timescale for the magnetization switching, expressed as (Δ*ω*)^−1^, can be ≈(2*αK*)^−1^ *ħ*. Here, Δ*ω* and *α* are spectral linewidth and effective damping constant, respectively. Generally, the timescale is longer than 1 ns (e.g., *ω* = 10 GHz and *α* = 0.1).

To consider collective spins in a chiral AF metal (Supporting Information 1), the following Hamiltonian to treat the inverse triangular spin structure can be used^[^ ^38^ ^]^

(1) $$H=J\sum_{\left\langle ia,jb\right\rangle }S_{ia}\cdot S_{jb}+D\sum_{\left\langle ia,jb\right\rangle }\varepsilon_{ab}z\cdot (S_{ia}\times S_{jb})-\frac{K}{2}\sum_{ia}{\left(k_{a}\cdot S_{ia}\right)}^{2}$$

Here, **S**, *J*, and *D* denote spin‐angular momentum, exchange interaction, and Dzyaloshinskii–Moriya interaction, respectively. (*i, j*) and (*a, b*) refer to the Mn sites and one of the three sublattices (*A*, *B*, and *C*) of the inverse triangular lattice structure, respectively. *K* is introduced to describe six‐fold magnetic anisotropy in Mn_3_Sn with **k**_a_ = (cos*ψ*_a_, sin*ψ*_a_, 0) and (*ψ*_A_, *ψ*_B_, *ψ*_C_) = (0, 4*π*/3, 2*π*/3). *ε*_ab_ is the antisymmetric tensor which satisfies *ε*_AB_ = *ε*_BC_ = *ε*_CA_ = 1 and **z** is the unit vector along the *c*‐axis. Note that in‐plane and out‐of‐plane magnetic anisotropies from the kagome plane should be determined by *K* and *D*, respectively. Figure 1d,e schematically show spin‐wave modes for a chiral AF metal. Mode I is an in‐plane (*xy*) optical mode. Although out‐of‐plane precession (*z*) is in‐phase and is governed by *D*, in‐plane precession is out‐of‐phase and is determined by *J*. Therefore, a typical energy scale (*ħω*_I_) of the mode I can be estimated as ≈$\sqrt{JD}$. Mode II is collective precession‐like mode, that is, in‐plane acoustic mode. Similarly, a typical energy scale (*ħω*_II_) of the mode II can be $\approx \sqrt{KJ}$. As we discuss in detail in the following section, although the resonant frequencies of modes I and II are different, typical timescales for the magnetization switching are identical, (Δ*ω*_I_)^−1^ ≈ (Δ*ω*_II_)^−1^ ≈ (*αJ*)^−1^ *ħ*, and thus is much shorter by the factor of *K*/*J* than the ferromagnetic case. Such an *exchange‐enhanced* ultrafast damped precession should be available in both AF and ferrimagnetic metals and would be the most significant in a fully compensated case. However, such compensation of magnetic moments has been considered to make it impossible to observe the spin‐dynamics in AF metals. This is because a signal amplitude is usually proportional to the net spin‐magnetization in a detection method for the spin‐dynamics, e.g., the Faraday effect and the MOKE. Here, we demonstrate the time‐resolved ultrafast spin precession in the AF Weyl semimetal Mn_3_Sn by using MOKE induced by the magnetic octupole order. The spin precessions from the noncollinear texture induce the oscillations of MOKE and the Berry curvature in the momentum space.

## 3 Results

### 3.1 Static MOKE

**Figure 2:**
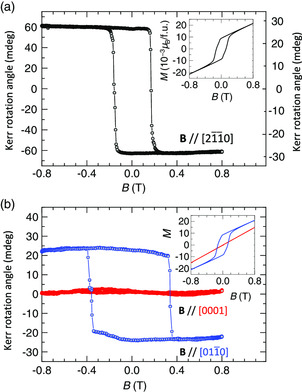
Static hysteresis loops of the MOKE. a) Magnetic field dependence of the polar MOKE result. b) Magnetic field dependence of the longitudinal MOKE results. Left (right) vertical axis shows Kerr rotation angle measured with 660 nm continuous‐wave (800 nm pulse) laser system. Insets show the corresponding magnetization hysteresis (*M* in 10^−3^ μ_B_ per f.u.) obtained as a function of the magnetic field with the same configuration as the MOKE in the main panel. MOKE signal does not come from the spin‐magnetization due to canting but from cluster magnetic octupole as discussed in the main text and in the study by Higo et al.^[^ ^31^ ^]^

In this article, bulk single‐crystal *D*0_19_‐Mn_3_Sn has been used (the Experimental Section). Mn_3_Sn has the hexagonal Ni_3_Sn‐type crystal structure consisting of an *ABAB* stacking of the kagome lattice of Mn atoms along with the [0001] axis. Red (blue) circles in Figure 1a indicate Mn atoms in the *A*‐(*B*‐) plane of the kagome lattice. Below the Néel temperature of 430 K, an inverse triangular spin structure is stabilized by exchange and Dzyaloshinskii–Moriya interactions.^[^ ^10^, ^39^, ^40^ ^]^ The inverse triangular spin structure possesses a uniform negative vector chirality of the in‐plane Mn moments and is made of a ferroic ordering of cluster magnetic octupoles. The magnetic moments cant slightly in the (0001)‐plane and produce a small net spontaneous magnetization,^[^ ^41^ ^]^ and can be reversed by an external magnetic field. Note that the appearance of the AHE,^[^ ^10^ ^]^ ANE,^[^ ^13^, ^14^ ^]^ and MOKE^[^ ^31^ ^]^ is not induced by the spin‐magnetization due to the canting but by the cluster magnetic octupole. As shown in **Figure** **2**a, the static MOKE signal has been confirmed in our crystal. Significantly, the polar MOKE signal, where the magnetic field is applied parallel to the kagome plane (***B*** // $\left[2\bar{1}\bar{1}0\right]$), exhibits a clear hysteresis with a large Kerr rotation angle (≈60 mdeg). This is three times larger than the previous report,^[^ ^31^ ^]^ and the difference may come from the optical interference effect.^[^ ^42^ ^]^ In addition to the polar MOKE, the longitudinal signals, where the magnetic field is applied in $\left[01\bar{1}0\right]$ and [0001] directions, were also characterized (Figure 2b). Although a clear hysteresis curve was observed in $\left[01\bar{1}0\right]$, no signal was confirmed in [0001]. The observed magnetic anisotropy is consistent with the previous works, where the octupole polarization in *D*0_19_‐Mn_3_Sn was detected via the measurements of the AHE,^[^ ^10^ ^]^ ANE,^[^ ^13^ ^]^ and MOKE.^[^ ^31^ ^]^ The right (left) vertical axis in Figure 2 shows the polar Kerr rotation angle measured with 660 nm continuous‐wave (800 nm pulse) laser system. The difference in the Kerr rotation angle obtained by the 660 nm and the 800 nm laser systems is consistent with the MOKE spectroscopy reported previously.^[^ ^31^ ^]^ Insets show magnetization hysteresis curves measured with the same magnetic field configuration as the MOKE measurements. From Figure 2a inset, the spontaneous magnetization of Mn_3_Sn is 9 × 10^−3^ μ_B_ per formula unit (f.u.). If we assume a conventional ferromagnet (e.g., Fe, Co, and Ni), a possible Kerr rotation angle at zero magnetic field from the spontaneous magnetization would be as small as 0.2 mdeg with positive polarity.^[^ ^31^ ^]^ Therefore, a large negative Kerr rotation angle at zero magnetic field can be hardly explained by the spin‐magnetization due to canting.

The octupole has the same irreducible representation of *T*_1*g*_ as the spin‐magnetization, and can induce the MOKE. The results of multipole expansion of the AF structure of Mn_3_Sn show that the octupole contributes to the expansion by more than 99.9%.^[^ ^32^ ^]^ The previous first‐principles calculation finds that the Kerr rotation angle has nearly no contribution from the net magnetization.^[^ ^31^ ^]^ Moreover, a method to obtain a low‐energy effective model of Mn_3_Sn has been recently developed based on the cluster multipole theory.^[^ ^38^ ^]^ The effective model presented in the study by Nomoto and Arita^[^ ^38^ ^]^ well reproduces the results for the domain wall dynamics and for the coherent steady precession of spins obtained by using the original spin Hamiltonian (Equation (1)).

### 3.2 Time‐Resolved MOKE

**Figure 3:**
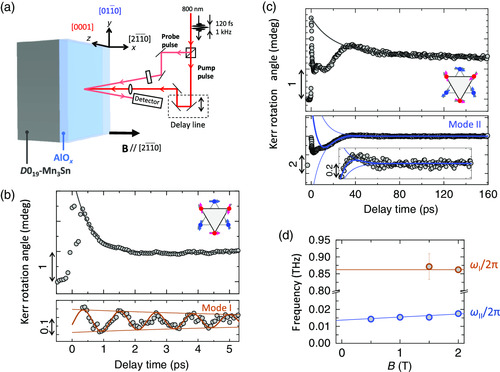
Time‐resolved oscillation of cluster magnetic octupole. a) Schematic diagram of the time‐resolved MOKE measurements (TR–MOKE). b,c) TR–MOKE signals of Mn_3_Sn under a magnetic field of 2 T in the $\left[2\bar{1}\bar{1}0\right]$ direction. Backgrounds, indicated by black curves in upper panels, were subtracted from the raw data and plotted in the lower panels. Orange (blue) thick curve corresponds to the fit, which yields the resonant frequency and effective damping constant, *ω*_I(II)/_2*π* = 0.86 THz (18 GHz) and *α*_I (II)_ = 0.02 (1.0), respectively. d) Magnetic field dependence of the oscillation frequency for the modes I (orange open circle) and II (blue open circle).

Our measurement of the time‐resolved MOKE (TR–MOKE) was made by using an all‐optical pump–probe method at room temperature (**Figure** **3**a and the Experimental Section). This method has been conventionally used to detect spin precession for ferromagnetic metals^[^ ^43^ ^]^ via Kerr effect and AF insulators^[^ ^35^, ^36^, ^37^ ^]^ via the Faraday effect. For an AF metal, however, no report on time‐resolved spin precession has been made to date. Pump and probe lights were configured almost perpendicular to the Mn_3_Sn $\left(2\bar{1}\bar{1}0\right)$‐surface. In addition, an external magnetic field normal to the surface (// $\left[2\bar{1}\bar{1}0\right]$) was applied during the measurements to direct the octupole polarization.

Figure 3b shows typical TR–MOKE results, where a magnetic field of 2 T was applied normal to the surface. First, a pump light induces a rapid increase in the Kerr rotation angle of the probe light. Because Mn_3_Sn is metallic, a pump pulse rapidly increases the electron temperature of the system. This rapid increase in electron temperature causes a significant decrease in the size of the order parameter, namely, the cluster magnetic octupole, similar to the case in ultrafast demagnetization in ferromagnetic metals.^[^ ^43^, ^44^ ^]^ After the excitation, a coherent spin precession starts with the aid of an effective magnetic field. As the cluster magnetic octupole is the order parameter that induces the MOKE signal as we discussed earlier, the spin precession is observed as the fluctuation of the cluster magnetic octupole. In Figure 3b, the MOKE intensity starts to recover with delay time more than 0.3 ps, exhibiting a small but clear oscillation during the recovery. To further characterize the oscillating component, a nonoscillating component was estimated as a background (black solid curve) and subtracted from the raw data (Supporting Information 2). The lower panel of Figure 3b shows the TR–MOKE signals after subtracting the background. Figure 3c also shows typical TR–MOKE results (upper panel) and the analysis for the oscillating component (lower panel) in a relatively long‐time range. The orange (blue) thick curve in the lower panel of Figure 3b (3c) corresponds to the fitting to the equation, cos(*ω*_I(II)_ *t* + *ϕ*_0_) exp(−*α*_I(II)_ *ω*_I(II)_ *t*), which yields both the resonant frequency and effective damping constant at *B* = 2 T, i.e., *ω*_I(II)/_2*π* = 0.86 THz (18 GHz) and *α*_I (II)_ = 0.02 (1.0), respectively. The orange (blue) thin curves represent the envelope functions of the fit, ±exp(−*α*_I(II)_ *ω*_I(II)_ *t*). Here, *t* is the delay time. The oscillation frequencies (*ω*_I_, *ω*_II_) as a function of the external magnetic field are shown in Figure 3d, which will be discussed later in detail. The oscillating behavior is most significant when the magnetic field is normal to the Mn_3_Sn surface. Because the TR–MOKE signal is proportional to the surface normal polarization of the cluster magnetic octupoles, the oscillating Kerr signal should come from a change of size in the octupole order parameter. The change of size in the octupole order parameter originates from the spin precession of each magnetic moment. Specifically for the mode I, the signal‐to‐noise ratio of the TR–MOKE significantly reduces with decreasing the magnetic field strength below 2 T. This is because an external magnetic field is necessary to create an effective magnetic field to drive a coherent precession (Supporting Information 3).

## 4 Discussion

Two oscillation modes I and II are found through the analyses shown in Figure 3b,c, which should come from the oscillation of the cluster magnetic octupoles (Supporting Information 2). To analyze the modes, we have derived the analytical solution to estimate the resonant frequencies from Equation (1). Note that Equation (1) includes the in‐plane exchange interaction *J* in the kagome lattice, but not the interplane exchange interaction, and thus is only valid for the modes where precessional motions in different kagome planes in‐phase. The equation of motion for low energy magnetic excitation can be derived from Equation (1) as the following sine‐Gordon equation (Supporting Information 4)

(2) $$\frac{\hbar }{2\sqrt{3}\left(D+\sqrt{3}J\right)S}\ddot{\phi }+\alpha \dot{\phi }-\frac{\left(\sqrt{3}D+J\right)S}{2\hbar }a_{\text{lat}}^{2}\partial^{2}\phi +\frac{KS}{2\hbar }\sin2\phi =0$$

where *ħ*, *α*, and *a*_lat_ indicate the reduced Planck constant, damping constant, and the lattice constant of the nearest neighbor Mn atoms, respectively. Here, *S* is the size of spin angular momentum ≈1.5 for the Mn magnetic moment ≈3 μ_B_, *ϕ* refers to in‐plane precession angle of Mn magnetic moment, and *α* is identical to the Gilbert damping constant in the Landau–Lifshitz–Gilbert equation. From Equation (2), resonant frequencies for modes I and II can be estimated as follows

(3) $$\hbar \omega_{I}=S\sqrt{6\sqrt{3}(\sqrt{3}D+J)D}$$

(4) $$\hbar \omega_{\text{II}}=S\sqrt{2\sqrt{3}K(D+\sqrt{3}J)}$$

Here, the resonant frequencies for the modes I and II (*ω*_I_ and *ω*_II_) correspond to the optical and collective precession‐like modes shown in Figure 1d,e, respectively. The damped oscillation is often expressed by exp(−*α*_I(II)_ *ω*_I(II)_ *t*) by introducing a phenomenological effective damping constant (*α*_I_, *α*_II_), which is expressed as

(5) $$\hbar \omega_{I}\alpha_{I}=\hbar \omega_{\text{II}}\alpha_{\text{II}}=2\sqrt{3}S(D+\sqrt{3}J)\alpha$$

Note that the effective damping (*α*_I_, *α*_II_) is not identical to the Gilbert damping constant (*α*) defined in Equation (2). The Gilbert damping constant should be determined from the damping rate (Equation (5)), which is independent of the resonant frequency (*ω*_I_, *ω*_II_). Although these two damping constants are almost identical for the case of a ferromagnet, a large difference between *ω*_I_ and *ω*_II_ induces large deviation of the effective damping constant from the Gilbert damping constant in Mn_3_Sn. This is a unique property of the octupole oscillation dynamics in a chiral AF metal and should be distinguished from spin‐magnetization dynamics in a ferromagnet. Recently, related discussion for the damping has been made in the magnetic domain‐wall dynamics in the ferrimagnetic GeFeCo.^[^ ^45^ ^]^ Here, the field dependence of the resonant frequencies (Figure 3d) is understood as follows. The resonant frequency of the optical mode (*ω*_I_) is determined by interactions between Mn atoms, and thus is insensitive to an external magnetic field. However, the resonant frequency of the collective precession‐like mode (*ω*_II_) is not. This is because the cluster magnetic octupole can couple with an external magnetic field via spontaneous magnetization due to canting. From the field dependence of the resonant frequencies (Figure 3d), *ω*_II/_2*π* at *B* = 0 is determined to be 13.7 ± 1.5 GHz.

**Figure 4:**
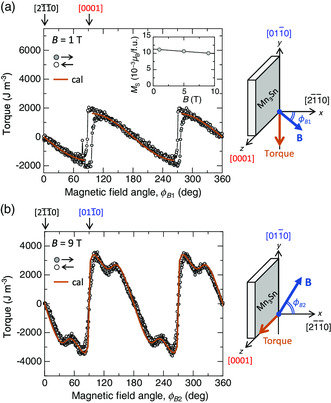
Magnetic anisotropy. a) Results of the torque measurements for the out‐of‐plane magnetic field rotation, namely, upon changing the direction of the magnetic field *B* from $\left[2\bar{1}\bar{1}0\right]$ (*ϕ*_B1_ = 0°) to [0001] (*ϕ*_B1_ = 90°). The inset shows the saturation magnetization (*M*_S_) as a function of an external magnetic field *B*. b) Results of the torque measurements for the in‐plane magnetic field rotation, namely, upon changing the direction of the magnetic field *B* from $\left[2\bar{1}\bar{1}0\right]$ (*ϕ*_B2_ = 0°) to [0001] (*ϕ*_B2_ = 90°). Solid and open circles represent the torque obtained with increasing and decreasing rotation angles of *ϕ*_B1_ and *ϕ*_B2_.

To characterize the dynamics of the cluster magnetic octupole, the six‐fold magnetic anisotropy *K* was determined from the torque measurements (Supporting Information 5). **Figure** **4**a shows the result of out‐of‐plane rotation (*y*‐axis) in terms of the plane consisting of the kagome lattice in Mn_3_Sn (*xy*‐plane) by rotating the direction of the magnetic field (**B**) from $\left[2\bar{1}\bar{1}0\right]$ (*ϕ*_*B*1_ = 0°) to [0001] (*ϕ*_*B*1_ = 90°). The solid and open circles indicate different rotation directions. The results allow the estimation of the saturation magnetization (*M*_S_), as shown in the inset of Figure 4a. The saturation magnetization should correspond to the spontaneous magnetization due to spin canting at zero field, determined by *D* and *J*, and should be distinguished from the magnetization components due to the spin canting induced by the application of an external magnetic field. Here, the saturation magnetization is estimated to be 11.3 × 10^−3^μ_B_ (10.0 × 10^−3^ μ_B_) per f.u. at *B* = 0 T (9 T), and is comparable to the magnetization at *B* = 0 (9 × 10^−3^ μ_B_ per f.u. from Figure 2a inset). In contrast, the in‐plane rotation (*z*‐axis) measurements of the torque were made by rotating the direction of the magnetic field (**B**) from $\left[2\bar{1}\bar{1}0\right]$ (*ϕ*_B2_ = 0°) to $\left[01\bar{1}0\right]$ (*ϕ*_B2_ = 90°) within the kagome‐lattice plane (Figure 4b). The analysis of the result estimates an energy barrier height from the six‐fold in‐plane magnetic anisotropy energy (*K*_6_/18, Supporting Information 5) to be =3.1 × 10^2^ Jm^−3^, consistent with the value reported in the previous work (2.2 × 10^2^ Jm^−3^).^[^ ^46^ ^]^ Here, the obtained in‐plane magnetic anisotropy energy corresponds to *K* = 3.1 × 10^−4^ meV from Equation (1).

In principle, six spin‐wave modes should be confirmed in Mn_3_Sn as the unit cell consists of six Mn atoms. Previous neutron scattering studies^[^ ^47^, ^48^ ^]^ have revealed the three modes in the low energy region (*ħω* < 20 meV) and the other three modes in the high energy region (*ħω* ≈ 100 meV) at **q** = 0, where **q** is the momentum vector. The low and high energy modes are the ones where precessional motions in different kagome‐planes are in‐phase and out‐of‐phase, respectively. Here, both the modes I and II in this article correspond to the low energy modes. From TR–MOKE results (Figure 3b), the energy of the mode I is 3.6 meV (0.86 THz). However, this is only 26% of the value obtained in the neutron scattering (≈14 meV).^[^ ^47^ ^]^ This strongly suggests that the size of the magnetic moment (see *S* in Equation (3)) is reduced. This might be because laser heated the sample close to its Néel temperature (∼430 K),^[^ ^39^ ^]^ which is not so far from room temperature, during the TR‐MOKE measurements. In fact, it has been reported that the AHE and MOKE are significantly suppressed by heating Mn_3_Sn.^[^ ^49^ ^]^ When *S* ≈ 0.4 (=1.5 × 26%) is used in Equation (3) and (4), *J* and *D* are estimated to be ≈10 and ≈0.7 meV, respectively, which is in good agreement with the previous studies.^[^ ^47^, ^48^, ^50^ ^]^ Gilbert damping constant of Mn_3_Sn, which is estimated for the first time, is found to be *α* = 0.0005 from Equation (5). The Gilbert damping constant of Mn_3_Sn is as small as the theoretical prediction for Mn–Ge^[^ ^51^ ^]^ and Mn–Ga^[^ ^52^ ^]^ alloys (0.0005–0.001).

As discussed, the dynamics characteristic of the cluster magnetic octupoles is an ultrafast damped oscillation due to the exchange interaction (Equation (5)). Interestingly, the exchange interaction dramatically increases the effective damping (*α*_II_ = 1.0). Thus, the typical switching time can be ultrafast (1/*ω*_I_ *α*_I_ ≈ 1/*ω*_II_ *α*_II_ ≈ 9 ps) although the resonant frequency for the collective precession‐like motion is relatively slow (*ω*_II_/2*π* = 13 GHz). As discussed, *S* ≈ 0.4 was used for the aforementioned analysis. If the sample temperature can be held much lower than the Néel temperature (*S* = 1.5), the switching time would be reduced to ≈2 ps. From the sine‐Gordon equation (Equation (2)), the Néel type‐domain wall velocity in Mn_3_Sn can be estimated as fast as 3 km s^−1^ (12 km s^−1^) for *S* = 0.4 (1.5) (Supporting Information 6), which is greater than the recently reported value for the ferrimagnetic systems in the vicinity of the angular momentum compensation temperature.^[^ ^53^, ^54^, ^55^ ^]^

For device applications, not only dynamic but also static properties of the cluster magnetic octupoles would be important. As discussed, energy barrier height (*K*_6_/18) for the six‐fold magnetic anisotropy energy of Mn_3_Sn is 3.1 × 10^2^ J m^−3^, which corresponds to the in‐plane magnetic anisotropy field of 3.8 T (=*K*_6_/*M*_S_). If we employ 10 nm thick Mn_3_Sn, the areal magnetic anisotropy energy should correspond to 3 μJ m^−2^. To obtain a thermal stability factor of 60 at 300 K, which is defined as magnetic anisotropy energy divided by thermal fluctuation energy (*k*_B_ *T*), relatively large magnetic cell as 320 nm in diameter (8 × 10^5^ nm^3^) is needed. However, such small magnetic anisotropy energy ensures that the octupole polarization can be efficiently controlled by electric current‐ or voltage‐driven torque.^[^ ^56^ ^]^

## 5 Conclusion

To conclude, our work demonstrates the giant effective damping of the octupole dynamics in an AF Weyl semimetal. The exchange interaction significantly increases the damping rate for the collective precession‐like mode, which is directly related to a typical switching time in device operation.^[^ ^57^ ^]^ The introduction of the key concept of *cluster magnetic octupole*, instead of *spin‐magnetization* in ferromagnets, has provided the basis for carrying out the experiments by using the conventional schemes developed for ferromagnetic spintronics. Thus, our observation will certainly foster the development of the topological spintronics.

## 6 Experimental Section

6.1

6.1.1

##### Sample Preparation

Polycrystalline samples were prepared by melting the mixtures of Mn and Sn in an Al_2_O_3_ crucible sealed in an evacuated quartz ampoule in a box furnace at 1050 °C for 6 h. In preparation for single‐crystal growth, the obtained polycrystalline materials were crushed into powders, compacted into pellets, and inserted into an Al_2_O_3_ crucible that was subsequently sealed in an evacuated SiO_2_ ampoule. Single‐crystal growth was performed using a single‐zone Bridgman furnace with a maximum temperature of 1080 °C and growth speed of 1.5 mm h^−1^. These were exactly the same methods for fabricating the single crystals as those used for the previous study on the Kerr effect^[^ ^31^ ^]^ and the chiral anomaly due to Weyl fermions.^[^ ^15^ ^]^ Analysis using inductively coupled plasma spectroscopy showed that the composition of the single crystal was Mn_3.07_Sn_0.93_. The bulk‐Mn_3_Sn sample was cut and polished so that the sample had optically smooth surfaces along the $\left(2\bar{1}\bar{1}0\right)$ plane. Then, the sample was annealed at 600 °C under vacuum (≈2 × 10^−6^ Pa) for 1 h. Without breaking vacuum, 4 nm AlO_*x*_ was subsequently prepared onto the Mn_3_Sn surface by electron beam deposition method to prevent degradation during MOKE measurements.

##### TR–MOKE Measurements

Time‐resolved polar MOKE signals were measured in a conventional all‐optical pump–probe setup using a Ti–sapphire laser with a regenerated amplifier.^[^ ^58^ ^]^ The incident light was almost perpendicular to the Mn_3_Sn surface, and the polar MOKE signal was proportional to the cluster magnetic octupole component normal to the Mn_3_Sn surface. The laser wavelength, pulse width, and repetition rate were 800 nm, 120 fs, and 1 kHz, respectively. A penetration depth of the incident light was ≈20 nm. The pump beam was modulated with a frequency of 360 Hz using an optical chopper, and the signal was detected using a lock‐in amplifier. Spot size and averaged laser power were ≈1 mW and *ϕ*0.66 mm for pump, and 0.05 mW and *ϕ*0.14 mm for probe beams, respectively. The delay time dependence of the Kerr rotation signal was recorded. The Kerr rotation signal was detected by the differential method. For the case of the TR–MOKE measurements, nonmagnetic backgrounds (e.g., a quadratic background confirmed in the static MOKE results (Figure 2a in the main text) attributed to an artifact from measurement setup) were cancelled by averaging the data measured for the reversed magnetic field. All measurements were performed at room temperature.

## Conflict of Interest

The authors declare no conflict of interest.

## Data Availability Statement

The data that support the findings of this study are available from the corresponding author upon reasonable request.

## Supporting information

Supplementary Material
