# Supplementary material for: Giant Effective Damping of Octupole Oscillation in an Antiferromagnetic Weyl Semimetal
Source: Small Sci. 2021 Apr 15;1(5):2000062. doi: 10.1002/smsc.202000062 (PMC11935827; doi:10.1002/smsc.202000062)
Supplement: Supplementary file 1 — Supplementary Material [file SMSC-1-2000062-s001.pdf]

Supporting Information

Giant effective damping of octupole oscillation in an antiferromagnetic Weyl semimetal

Shinji Miwa, Satoshi Iihama, Takuya Nomoto*, Takahiro Tomita, Tomoya Higo, Muhammad Ikhlas, Shoya Sakamoto, YoshiChika Otani, Shigemi Mizukami, Ryotaro Arita, and Satoru Nakatsuji*

1. Resonant frequency and spectral linewidth

When we treat the spherical coordinate system (ϑ, ϕ) to describe spin-direction, resonant frequency (ω) and spectral linewidth ($\Delta\omega$) can be derived as follows from Landau-Lifshitz-Gilbert equation,[S1]

$$\omega \approx \sqrt{\det\left[\hat{\Omega}\right]} \approx \sqrt{\omega_{\phi\phi}\omega_{\theta\theta}}\ , \qquad \text{(S1)}$$

$$\Delta\omega \approx \mathrm{Tr}\left[\hat{\Delta}\right] = \Delta\omega_{\phi\phi} + \Delta\omega_{\theta\theta}, \quad \text{(S2)}$$

$$\hat{\Omega} \equiv \begin{pmatrix} \omega_{\phi\phi} & \omega_{\phi\theta} \\ \omega_{\theta\phi} & \omega_{\theta\theta} \end{pmatrix} = \frac{1}{\left(1+\alpha^2\right)} \begin{pmatrix} \dfrac{\partial^2 U}{\partial \phi^2} & -\dfrac{1}{S\sin\theta}\dfrac{\partial^2 U}{\partial\theta\partial\phi} \\ -\dfrac{1}{S\sin\theta}\dfrac{\partial^2 U}{\partial\theta\partial\phi} & \dfrac{1}{S\sin\theta}\dfrac{\partial}{\partial\theta}\dfrac{1}{S\sin\theta}\dfrac{\partial U}{\partial\theta} \end{pmatrix}, \qquad \text{(S3)}$$

$$\hat{\Delta} \equiv \begin{pmatrix} \Delta\omega_{\phi\phi} & \Delta\omega_{\phi\theta} \\ \Delta\omega_{\theta\phi} & \Delta\omega_{\theta\theta} \end{pmatrix} = \frac{\alpha}{S} \begin{pmatrix} \dfrac{\partial}{\partial\phi}\dfrac{1}{\sin^2\theta}\dfrac{\partial U}{\partial\phi} & - \\ - & \dfrac{1}{\sin\theta}\dfrac{\partial}{\partial\theta}\sin\theta\dfrac{\partial U}{\partial\theta} \end{pmatrix}. \qquad \text{(S4)}$$

Here, U, α, and S are potential energy, Gilbert damping constant and spin angular momentum, respectively.

For a ferromagnet where potential energy is determined by uniaxial magnetic anisotropy energy (K), $\omega \sim K\hbar^{-1}$ can be derived. This can be understood as both ϕ- and ϑ- precessional motions are in-phase and are governed only by K. Spectral linewidth can be $\Delta\omega_{\phi\phi} + \Delta\omega_{\vartheta\vartheta} \sim 2\alpha K\hbar^{-1}$ ($\because$ $\Delta\omega_{\phi\phi} = \Delta\omega_{\vartheta\vartheta} \sim \alpha K\hbar^{-1}$). Consider the mode I (optical mode) in a chiral AF metal, where kagome-plane is in ϕ-plane (xy-plane in Figure 1d in the main text). Here, the resonant frequency can be $\omega_{\mathrm{I}} \sim \sqrt{JD}\hbar^{-1}$ (see Eq. 3 in the main text for the exact solution). This is because out-of-phase ϕ-motion (xy-plane) is determined by exchange interaction (J) and in-phase ϑ-motion (z) is determined by Dzyaloshinskii-Moriya interaction (D), where D acts as the magnetic anisotropy for the ϑ-motion. Spectral linewidth can be $\Delta\omega_{\phi\phi} + \Delta\omega_{\vartheta\vartheta} \sim \alpha J\hbar^{-1}$ ($\because$ $\Delta\omega_{\phi\phi} \sim \alpha JS\hbar^{-1}$ and $\Delta\omega_{\vartheta\vartheta} \sim \alpha DS\hbar^{-1}$, see Eq. 5 in the main text for the exact solution). For the mode II (collective precession mode) in a chiral AF metal, the resonant frequency can be $\sim\sqrt{KJ}\hbar^{-1}$ (see Eq. 4 in the main text for the exact solution) because in-phase ϕ-motion and out-of-phase ϑ-motion are determined by K and J, respectively. Spectral linewidth can be

$\Delta\omega_{\phi\phi} + \Delta\omega_{\vartheta\vartheta} \sim \alpha J\hbar^{-1}$ ($\because \Delta\omega_{\phi\phi} \sim \alpha KS\hbar^{-1}$ and $\Delta\omega_{\vartheta\vartheta} \sim \alpha JS\hbar^{-1}$, see Eq. 5 in the main text for the exact solution).

2. Characterizing the resonant frequency and the effective damping constant.

To characterize the resonant frequency and the effective damping constant, the obtained signal was fit by an equation describing damped oscillation which is proportional to $\cos(\omega_{\mathrm{I(II)}}t+\phi)\exp(-\alpha_{\mathrm{I(II)}}\omega_{\mathrm{I(II)}}t)$, where $\omega_{\mathrm{I(II)}}$, ϕ and $\alpha_{\mathrm{I(II)}}$ are the resonant frequency, the initial phase, and the effective damping constant of modes I (II), respectively. Because the damped oscillation was confirmed during the relaxation from the ultrafast demagnetization-like behavior, an appropriate background expressing the relaxation process should be subtracted from the raw data. As a function to describe the background, we employed an exponential function, which is depicted in the upper panel of Figure 3b and 3c in the main text as a black solid curve.

Because of the large effective damping constant (α_{II} = 1.0), the oscillation cannot be recognized at a glance in Figure 3c in the main text. To increase the accuracy of the fit, a background signal, where there is no spin-wave-induced oscillation, is obtained by measuring a sputter-deposited polycrystalline 50 nm-Mn_3Sn film. Because the *c*-axis of each Mn_3Sn grain is randomly oriented in the film, coherent excitation should not be made. Here, the TR-MOKE experiment is done with the setup and conditions that are different from those employed in the main text using bulk-Mn_3Sn. The laser wavelength, pulse width, and repetition rate are 800 nm, 140 fs, 80 MHz, respectively. Pump fluence is ~10 times smaller than that employed in Figure 3 in the main text. Because of the relatively small Kerr rotation angle for the 800 nm-wavelength (Figure S1 inset), we have employed a different pulse laser system from the one employed in the main text. We employed magnetron sputtering to prepare a polycrystalline Mn_3Sn thin film and confirmed that the film exhibits anomalous Hall effect and MOKE signal due to the cluster magnetic octupole (Figure S1 inset). The sample fabrication procedure can be found elsewhere.[S2] As shown in Figure S1, TR-MOKE results indicate the absence of the oscillation signals attributed to mode II. The black solid curve shows a fit using an exponential function. The same background is used for the one in Figure 3c in the main text.

In Figure 3c of the main text, there is an oscillation-like signal around 10 ps, which is apparently different from the oscillation signals originating from the modes I and II. As explained above, TR-MOKE results in Figure S1 indicate the absence of the oscillation signals attributed to modes I and II but a similar oscillation-like signal around 10 ps remains. As this point, we do not have enough information to identify its origin. However, it should be noted that the signal has nothing to do with the coherent spin-wave oscillation.

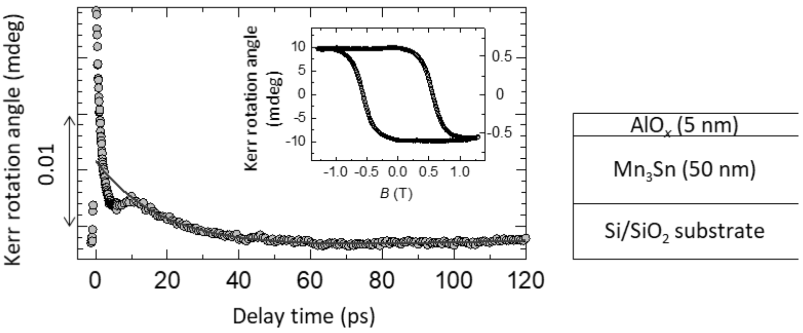

Figure S1. TR-MOKE results for a Mn_3Sn thin film. Randomly oriented polycrystalline 50nm-Mn_3Sn is employed for the TR-MOKE measurement. An external magnetic field of ±1.2 T normal to the film plane is applied during the measurements. The black solid curve shows the fit using an exponential function. The background for Figure 3c in the main text is based on this fit. Inset shows the hysteresis loops of MOKE due to the octupole polarization, where a magnetic field is applied normal to the film plane. The left (right) axis of inset shows the polar Kerr rotation angle measured with 660-nm continuous-wave (800-nm pulse) laser system.

3. Coherent spin-wave excitation in Mn_3Sn

During the TR-MOKE measurements, an external static magnetic field is applied to direct the octupole polarization along the magnetic field direction. Moreover, the static magnetic field is necessary to adjust the initial phase of each Mn spin to an identical one, which is indispensable for observation of mode I. Figure S2a shows a ground state of the spin structure of Mn_3Sn under zero-field while Figure S2b presents the configuration stabilized under an external magnetic field. As shown in Figure S2b, an external magnetic field induces the canting of the magnetic moments and aligns polarization of all the octupoles along with the same direction as the initial phase depicted in Figure 1d in the main text. When the parameters (*J*, *D*, and *K*) are decreased by a pump pulse, the canting angle increases, and the precession can be driven coherently.

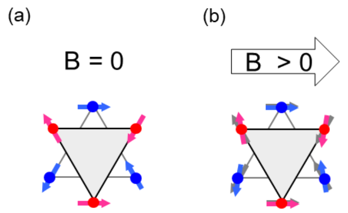

Figure S2. (a) Spin structure of Mn_3Sn under zero field. (b) Spin structure of Mn_3Sn under a static magnetic field.

Here, we consider a model to analyze the octupole oscillations in the TR-MOKE signal after the coherent excitation. As shown in Figure 2a in the main text, the Kerr rotation angle of the Mn_3Sn is negative as a response to the octupole polarization. Therefore, the oscillation should start with increasing the Kerr rotation angle when the phase delay ϕ_0 is negligible (see the dashed curves in Figure S3a for $\phi_0 = 0$ deg.). This can be understood as follows. A pump pulse induces a rapid change in the temperature and the magnetic moment, decreasing the anisotropy energy of each magnetic moment determined by the exchange and the spin-orbit interactions. Therefore, the pump pulse increases a spin-canting induced by an external magnetic field (**B**) as explained above. In other words, the pump pulse creates an effective magnetic field parallel to **B**, following the similar mechanism to the case of a ferromagnet.[S3] As a result, the torque to drive a coherent precession arises along out-of-kagome-plane direction as indicated in red (Figure S3a, see below). When the coherent precession starts, the octupole order parameter reduces. For the case of Figure 3b in the main text, the phase delay (ϕ_0) cannot be neglected because the transition time for the recovery (τ ~ 0.7 ps) is comparable to the oscillation period (~1.2 ps ($\omega_I/2\pi$=0.86 THz)). Here, let us assume the exponential decay of the torque on each magnetic moment (as shown in Figure S3b) because this is the simplest and most natural temporal profile of the torque. Then the phase delay can be derived to be $\phi_0 = \tan^{-1}(\omega_I\tau)$ as

depicted in Figure S3c.[S4] For the observed recovery time of $\tau = 0.7$ ps, a phase delay is estimated to be around 70-80 deg., which is close to the maximum one ($\phi_0 = 90$ deg.). Notably, after considering the phase delay, the simulated Kerr rotation angle (solid curve in Figure S3a) well reproduces the observed oscillation signal in Figure 3b in the main text.

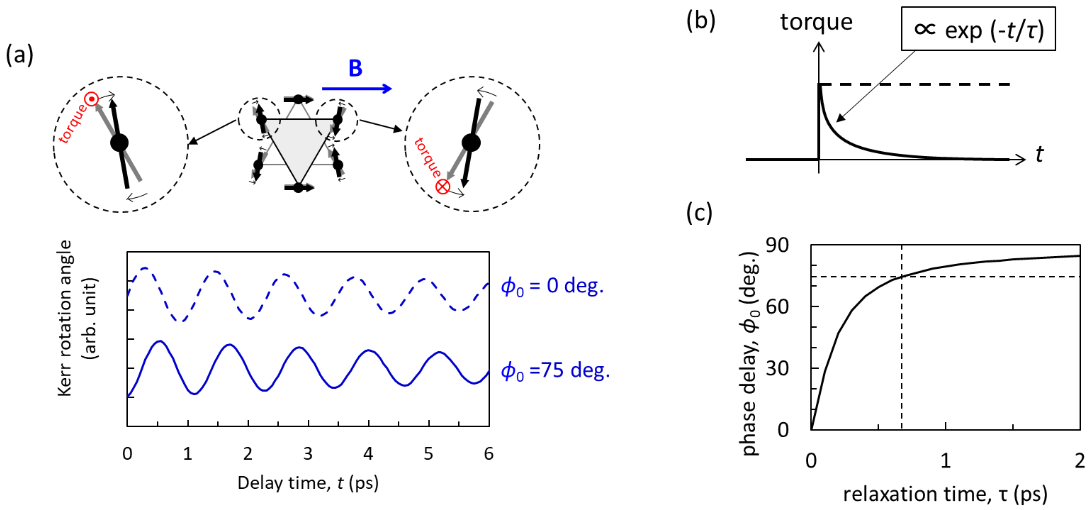

Figure S3. (a) Possible MOKE oscillation from the octupole polarization. (b) Temporal profile of a torque used for calculation of Figure S3c. (c) Calculated phase delay as a function of the relaxation time.

4. Theoretical study to determine the spin-wave modes

Spin-wave excitations in Eq. 1 were calculated on the inverse triangular spin structure. First of all, we transform the local frame such that its z-axis is parallel to the classical spin direction of the ground state.[S5] For the spin operator defined on the new frame ($\mathbf{S}'_{ia} = O_a^{-1}\mathbf{S}_{ia}$), we then perform the Holstein-Primakoff transformation ($\mathbf{S}'_{ia} = M\mathbf{a}_{ia}$), where O_a, M, and $\mathbf{a}_{ia}$ are respectively given by

$$O_a = \begin{bmatrix} 0 & -\sin\psi_a & \cos\psi_a \\ 0 & \cos\psi_a & \sin\psi_a \\ -1 & 0 & 0 \end{bmatrix}, \quad M = \sqrt{\frac{S}{2}} \begin{bmatrix} 1 & 1 & 0 \\ -i & i & 0 \\ 0 & 0 & \sqrt{\frac{2}{S}} \end{bmatrix}, \quad \text{(S5)}$$

and $\mathbf{a}_{ia} = (a_{ia},\ a^{\dagger}_{ia},\ S - a^{\dagger}_{ia}a_{ia})$. Here, the annihilation (creation) operator a_{ia} ($a^{\dagger}_{ia}$) represents the Holstein-Primakoff boson, and the Hamiltonian (Eq. 1 in the main text) is expressed in terms of a_{ia} and $a^{\dagger}_{ia}$. Within the linear spin-wave approximation, we neglect higher-order terms of a_{ia} and $a^{\dagger}_{ia}$, and calculate spin-wave excitation energies keeping them up to the quadratic order.

5. Analysis for the torque measurements

Figure S4 shows the schematic diagram of the detailed torque measurements setup of the in-plane magnetic field rotation in the kagome-lattice of Mn_3Sn. The setup corresponds to the experiment for Figure 4b in the main text. Here **B**, **m**, ϕ_B, and ϕ_m show the magnetic field vector, unit vector along with the spontaneous magnetization, magnetic field angle, and angle of the

spontaneous magnetization, respectively. Note that the direction of the spontaneous magnetization should be parallel to the octupole polarization. Measurements were conducted at 300 K. Orange curves in Figure 4a and 4b in the main text show calculated results to reproduce the torque experiments using the following equation:

$$\text{Torque: } -\left.\frac{\partial U}{\partial \phi}\right|_{\phi \to \phi_m}, \qquad \text{(S6)}$$

$$U = \frac{K_2}{2}\sin^2\phi + \frac{K_4}{8}\sin^2 2\phi + \frac{K_6}{18}\sin^2 3\phi, \qquad \text{(S7)}$$

where U represents magnetic anisotropy energy. Here, $K_2/2$ (= 3.4×10^3 $\mathrm{Jm^{-3}}$), $K_4/8$ (= -1.8×10^2 $\mathrm{Jm^{-3}}$), and $K_6/18$ (= 3.1×10^2 $\mathrm{Jm^{-3}}$) are the energy barrier heights for two-, four- and six-fold magnetic anisotropy. Therese values are comparable to those obtained in the previous work.[S6]

From the crystal structure of $D0_{19}$-Mn_3Sn, only six-fold magnetic anisotropy should appear. However, we find that in addition to the K_6 term, K_2 and K_4 terms are indispensable to reproduce the experimentally obtained torque data and are strongly field-dependent. Therefore, K_2 and K_4 terms would be attributed to magnetostriction in Mn_3Sn, and an intrinsic magnetic anisotropy at **B** = 0 should be six-fold only, being described by $K_6/18$ = 3.1×10^2 $\mathrm{Jm^{-3}}$. Thus, the energy height ($K_6/18$) determines the thermal stability of the octupole polarization domain at **B** = 0. Because the K_6 term corresponds to S^2K in Eq. 1 in the main text, K is calculated to be 3.1×10^{-4} meV per Mn atom with S = 1.5.

The saturation magnetization (M_S) of Mn_3Sn should correspond to the spontaneous magnetization due to spin canting at zero field, determined by D and J, and should be distinguished from the magnetization components due to the spin canting induced by the application of an external magnetic field. From the out-of-plane magnetic field rotation measurements (Figure 4a in the main text), the saturation magnetization can be characterized under the assumption of $M_S B \ll K_2$. From the inset of Figure 4a in the main text, M_S was estimated be $11.3\times10^{-3}\mu_B$ per f.u. at **B** = 0.

To direct the octupole polarization in a single-domain Mn_3Sn from the easy (e.g. $[2\bar{1}\bar{1}0]$) to hard (e.g. $[01\bar{1}0]$) axes, the magnetic field of 3.8 T (=K_6/M_S) should be necessary. In a bulk sample, a much smaller magnetic field should be enough to conduct magnetization switching as shown in Figure 2a inset in the main text because the magnetization switching can be done via a domain wall sweep.

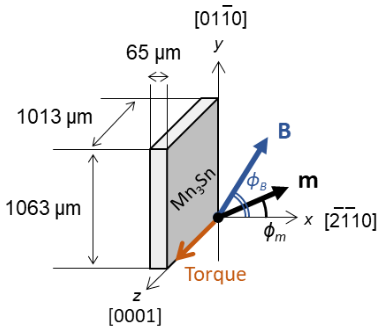

Figure S4. Measurement configuration for torque measurements. The schematic diagram of the detailed torque measurements setup of the in-plane magnetic field rotation in the kagome-lattice plane of Mn_3Sn.

6. Maximum domain wall velocity of the octupole polarization

The maximum domain wall velocity (v_{max}) can be derived from Eq. 2 in the main text, where $\alpha = 0$, $K = 0$, $\phi \sim \exp(i\omega t - ikx)$, and $\omega = v_{max}k$ are employed.[S7]

$$v_{\max} = \frac{a_{\mathrm{lat}}}{\hbar} S \sqrt{\left(\sqrt{3}D + J\right)\left(\sqrt{3}D + 3J\right)} \quad \text{(S8)}$$

For the case of Mn_3Sn, the domain wall propagates with a high velocity without showing the Walker breakdown because of strong magnetic anisotropy due to the Dzyaloshinskii-Moriya interaction, which prevents the out-of-kagome-plane motion (Figure 4a in the main text).[S7]

[S1] Y. Suzuki, *Spin current (Oxford University Press)* **2012**, 343.

[S2] T. Higo, D. Qu, Y. Li, C. L. Chien, Y. Otani, S. Nakatsuji, *Appl. Phys. Lett.* **2018**, *113*, 202402.

[S3] A. Kirilyuk, A. V. Kimel, T. Rasing, *Rev. Mod. Phys.* **2010**, *82*, 2731.

[S4] Schellekens, K. C. Kuiper, R. R. J. C. de Wit, B. Koopmans, *Nat Commun* **2014**, *5*, 4333.

[S5] F. J. dos Santos, M. dos Santos Dias, F. S. M. Guimarães, J. Bouaziz, S. Lounis, *Phys. Rev. B* **2018**, *97*, 024431.

[S6] T. F. Duan, W. J. Ren, W. L. Liu, S. J. Li, W. Liu, Z. D. Zhang, *Appl. Phys. Lett.* **2014**, *107*, 082403.

[S7] T. Nomoto, R. Arita, *Phys. Rev. Research* **2020**, *2*, 012045(R).
